# A Constitutively Active Cytokinin Receptor Variant Increases Cambial Activity and Stem Growth in Poplar

**DOI:** 10.3390/ijms23158321

**Published:** 2022-07-28

**Authors:** Michael Riefler, Tobias Brügmann, Matthias Fladung, Thomas Schmülling

**Affiliations:** 1 Institute of Biology/Applied Genetics, Dahlem Centre of Plant Sciences, Freie Universität Berlin, Albrecht-Thaer-Weg 6, 14195 Berlin, Germany; rieflerm@zedat.fu-berlin.de; 2 Thuenen Institute of Forest Genetics, Sieker Landstraße 2, 22927 Großhansdorf, Germany; tobias.bruegmann@thuenen.de (T.B.); matthias.fladung@thuenen.de (M.F.)

**Keywords:** biomass, cambium, cytokinin, poplar, plant growth

## Abstract

The cambial meristem is responsible for bark and wood formation in woody plants. The activity of the cambial meristem is controlled by various factors; one of them is the plant hormone cytokinin. Here, we have explored different approaches to genetically engineering cambial activity in poplar plants by the ectopic expression of a cytokinin biosynthesis gene with enhanced activity (named *ROCK4*) or of a gene encoding a constitutively active cytokinin receptor variant (*ROCK3*). Both genes are derived from *Arabidopsis thaliana* and were expressed in poplar trees under the control of their own promoter or the cambium-specific *pHB8* promoter. *pIPT3:ROCK4*- and *pHB8:ROCK4*-expressing plants were smaller than wild-type plants and formed more lateral branches; *pHB8:ROCK4* transgenic plants additionally showed an increased stem diameter. In contrast, *pAHK3:ROCK3*- and *pHB8:ROCK3*-expressing plants grew taller than wild type without an altered branching pattern and formed more cambial cells, leading to increased radial stem growth. The effectivity of *ROCK3* when expressed in either secondary phloem cells or in cambial cells is consistent with a dual, tissue-autonomous and non-autonomous activity of cytokinin in regulating cambial activity. We propose *ROCK3* as a novel gene to enhance biomass formation in woody plants.

## 1. Introduction

Plant biomass is an important source of raw material and renewable energy, it is also a major sink for atmospheric carbon dioxide (CO_2_) [1,2]. In the light of ongoing climate change, trees are important producers of plant biomass, which is primarily stored in the secondary xylem (wood). Trees have significant advantages for biomass production as they need less energy than food crops to produce similar amounts of biomass, grow often on soils not used in agriculture, do not need fertilization, and do not compete with usage as food. Plantation forests including short rotation coppices cultures (e.g., for poplars) have been developed to meet the increasing demand for wood production [3] as they offer a decentralized, storable, and renewable energy source [4]. The optimization of biomass formation by trees is of high economic interest not only to produce renewable energy but also for its use in the production of biopolymers, for example in the pulping industry. 

The bulk of tree biomass is wood. Wood is formed by the vascular cambium, which is a circular bifacial secondary meristem in the stem [5]. It produces phloem on the outside and xylem on the inner side. Xylem is transformed into wood by the deposition of lignocellulosic fibers in the secondary walls. The activity of the vascular cambium is the primary factor determining the amount of wood, and thus of biomass, that is produced in trees. Therefore, the regulation of cambial activity has been intensively studied and numerous regulatory factors have been identified, including plant hormones [6]. One key regulatory factor of vascular cambium activity is the hormone cytokinin [7,8,9,10]. 

Cytokinin metabolism and signaling has been elucidated in *Arabidopsis thaliana* and the relevant genes have been identified (for review see: [11,12]). The orthologous genes are present in the genome of poplar plants [13,14,15]. The initial and often rate-limiting step of cytokinin biosynthesis is catalyzed by isopentenyltransferases (IPT) [16,17]. The reaction products are cytokinin nucleotides, which are metabolized further to form the biologically active free bases, principally isopentenyladenine and trans-zeatin. The cytokinin signal is perceived by membrane-located histidine kinases which feed into a two-component signaling system activating type-B response regulator proteins (RRBs), which are transcription factors mediating the primary transcriptional output of cytokinin signaling. Among the target genes of RRBs are type-A *RR* (*RRA*) genes. The expression level of *RRA* genes is often used experimentally as a record of the cytokinin status of a given tissue (e.g., [18,19,20]). Numerous *RRA* genes of poplar are also responsive to cytokinin [13]. 

Previous work has highlighted the role of cytokinin as a major positive regulator of cambial activity in Arabidopsis and in trees. A reduced cytokinin concentration due to mutation of cytokinin-synthesizing *IPT* genes or obtained by ectopic overexpression of cytokinin-degrading CKX enzymes leads to reduced radial growth in tobacco, Arabidopsis and poplar [7,8,18,21]. In contrast, increased cytokinin content and signaling stimulate cambial cell divisions and lignocellulosic biomass production [22,23,24]. Detailed analysis of cytokinin distribution showed that the cytokinin concentration is highest in the secondary phloem and gradually declines through the cambial meristematic zone and the adjacent xylem [8,24]. Consistently, the *IPT* genes of poplar plants show the highest expression in the developing phloem tissue [23] while expression of certain cytokinin receptor genes peak in the cambial zone [8,24].

The activity of cytokinin on cambial growth has been described as being composed of two parts [24]. One part is derived from the cytokinin-enriched secondary phloem, which produces an as yet unidentified non-cell-autonomous paracrine signal being transported to the neighboring cambial cells, where it promotes cell division. Consistently, the phloem-specific reduction of cytokinin decreases radial growth [24]. A second activity of cytokinin is located in the cambium itself where cytokinin receptor genes are expressed. The degradation of cytokinin in cambium and adjacent xylem reduced radial stem growth as well, supporting the direct action of cytokinin in this tissue [8,24]. Taken together, cytokinin signaling located in the secondary phloem and vascular cambium synergistically regulate vascular cambial activity.

Recently, novel tools to manipulate cytokinin biosynthesis and signaling have become available. Several genes possessing enhanced cytokinin synthesis or signaling activity have been isolated during a suppressor screen searching for mutants reverting the phenotypic consequences of cytokinin deficiency in Arabidopsis. One gene, named *REPRESSOR OF CYTOKININ DEFICIENCY4* (*ROCK4*), carries a mutation in the *IPT3* gene, rendering the encoded cytokinin-synthesizing IPT3 protein more active [25]. A second gene identified in that suppressor screen, *ROCK3*, carries a mutation in the ligand-binding domain of the ARABIDOPSIS HISTIDINE KINASE3 (AHK3) cytokinin receptor, causing its constitutive activation [26]. Thus, ROCK4 can be used to increase cytokinin biosynthesis and ROCK3 to achieve cell-autonomous enhanced cytokinin signaling. In Arabidopsis, the expression of the *ROCK4* and *ROCK3* genes under control of their own promoter led to a significant increase of the cell number of interfascicular cambium, and together with an increased cell size, an up to 50% greater stem diameter [25,26].

Here, we have explored the use of *ROCK4* and *ROCK3* to enhance cambial activity and thus radial growth in transgenic poplar plants. *ROCK4* expression would generate more of a mobile cytokinin signal whereas *ROCK3* would enhance cytokinin signaling only in those cells that express the gene. We expressed both genes under the control of their original Arabidopsis promoter and the cambial *pHB8* promoter and compared the growth of transgenic lines to wild-type plants. Ectopic expression of the *ROCK3* gene caused increased longitudinal and radial growth, highlighting enhanced cell-autonomous cytokinin signaling as a tool to increase cambial activity and stem growth in trees. 

## 2. Results 

### 2.1. Characterization of pIPT3:ROCK4 Transgenic Poplar Plants

**Figure 1:**
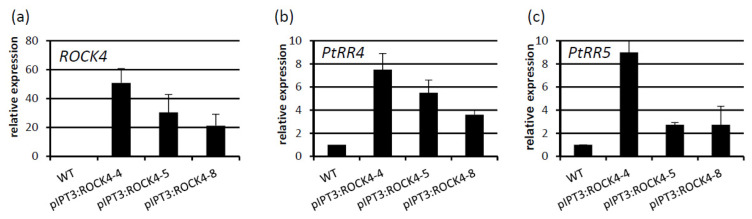
Expression of the *ROCK4* transgene and *PtRR* genes in shoots of *pIPT3:ROCK4* poplar plants grown in vitro. (**a**) Relative expression of *ROCK4* and the A-type response regulator genes (**b**) *PtRR4* and (**c**) *PtRR5*, measured in stem tissue four weeks after subcultivation of in vitro grown *pIPT3:ROCK4* transgenic poplar plants. In (**a**), the lowest level of transgene expression was set to 20. In (**b**,**c**), the expression of WT was set to 1. Data shown are mean values ± SD (*n* = 4).

At first we explored the usefulness of the *ROCK4* gene, which encodes a more active variant of the Arabidopsis cytokinin biosynthesis gene *IPT3*. *IPT3* is involved in regulating cambial activity in Arabidopsis [7]. *ROCK4* was positioned under control of the Arabidopsis *IPT3* gene promoter (*pIPT3:ROCK4*), which is principally active in phloem cells [27]. Among the eight primary transformants, three independent lines expressing *ROCK4* were identified and selected for further analysis (Figure 1a). The expression level of two cytokinin-responsive poplar *ARR* genes, *PtRR4* and *PtRR5* [13], were used as an indicator of the cytokinin status. qPCR analysis showed an approximately 4–8-fold higher mRNA level of *PtRR4* and an approximately 2–8-fold increase of *PtRR5*, indicating an increased cytokinin signaling output (Figure 1b,c). The differences in the relative increase of expression of *RRA* genes in the three lines reflected the differences in transgene expression. 

**Figure 2:**
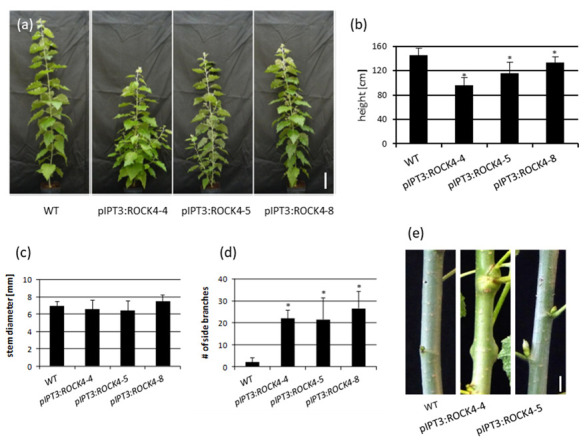
Phenotypical characterization of *pIPT3:ROCK4* transgenic poplar plants grown in a greenhouse. (**a**) Phenotype of 12-week-old wild type (WT) and pIPT33:ROCK4 plants. Bar size = 20 cm. (**b**) Plant height, (**c**) stem diameter 20 cm above ground and (**d**) number of side branches of 12-week-old plants. Data shown are mean values ± SD (*n* = 9–13). (**e**) Stems with axillary buds and side branches, leaves were removed. Bar size = 5 mm. Statistical significance of differences to wild type were calculated using Student’s *t*-test. *, *p* < 0.05.

After transfer to the greenhouse, plant height, stem diameter and branching were recorded in weekly intervals. Figure 2a shows that *pIPT3:ROCK4* lines had an approximately 10–40% reduction in plant height compared to wild-type poplar (Figure 2b). The mean stem diameter increased in only one of the three lines but was not significantly different from the wild type (Figure 2c). All transgenic lines showed a strong branching phenotype with more than 20 side branches that had formed 12 weeks after transfer to the green house (Figure 2d). Only the lateral buds at the base of the stem grew out to form longer branches while more apical buds formed short, slow-growing branches or stopped growing soon after the initial growth (Figure 2a,e).

Taken together, the ectopic expression of the *ROCK4* gene under control of the *IPT3* promoter causes morphological changes indicating an enhanced cytokinin status, which is confirmed by an increased cytokinin reporter gene expression. This shows that the *ROCK4* gene is functional in poplar, but the morphological alterations obtained using the *IPT3* promoter were not favorable. We, therefore, aimed in the next step to explore the consequences of *ROCK4* gene expression under control of a promoter with cambial expression. 

### 2.2. Expression of the ROCK4 Gene under Control of the HB8 Promoter

**Figure 3:**
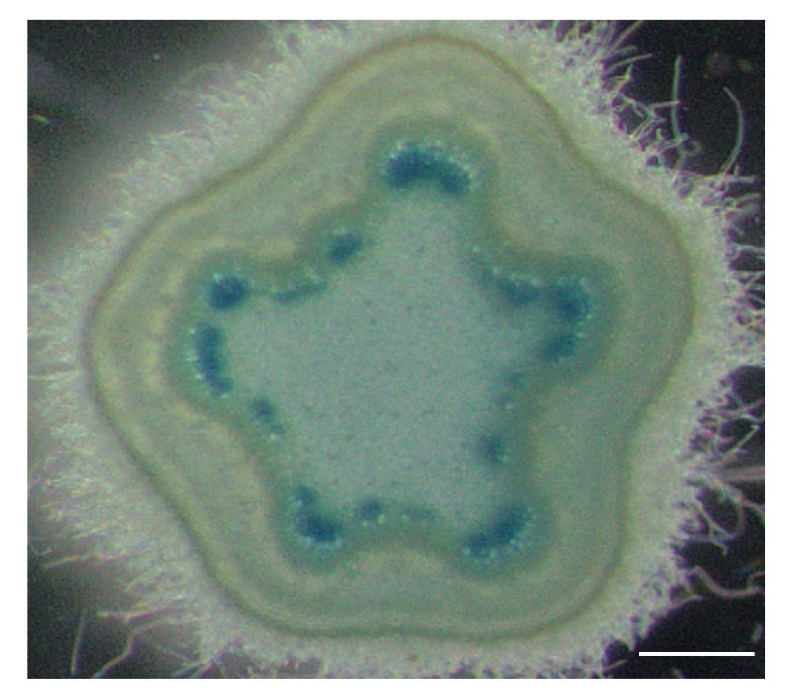
Expression of a *pHB8:GFP-GUS* reporter gene in stems of poplar plants. The figure shows a cross section of a stem of 4-week-old poplar plant grown in vitro and stained overnight, visualizing *pHB8:GFP-GUS* reporter gene expression in the cambium and developing xylem cells. Bar size is 100 µm.

We choose the *HB8* promoter of Arabidopsis, which is expressed in procambial and vascular cells [28,29] to drive *ROCK4* expression. To test first the expression of the promoter in poplar we constructed a *pHB8:GFP-GUS* reporter gene and monitored its expression in transgenic poplar plants. Transverse stem sections showed GUS staining in cambial cells and developing xylem (Figure 3) suggesting that the *HB8* promoter largely retains its expression characteristics in poplar. 

**Figure 4:**
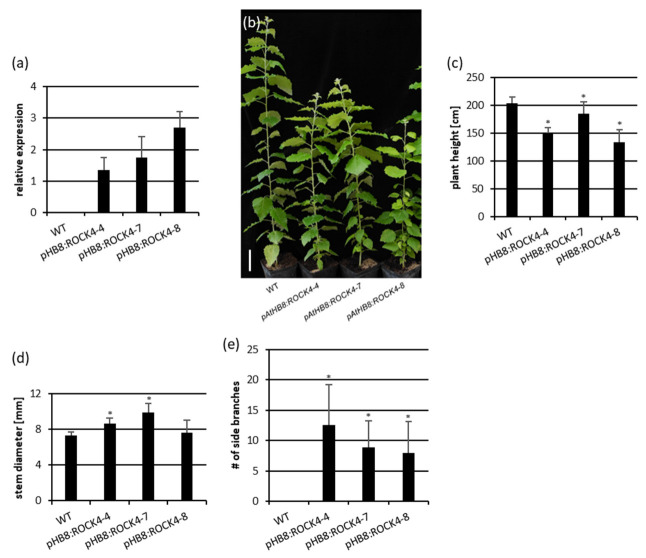
Characterization of *pHB8:ROCK4* transgenic poplar plants grown in vitro and in a greenhouse. (**a**) Relative expression of *pHB8:ROCK4* in stem tissue of plants grown in vitro. Samples were harvested four weeks after subcultivation. (**b**) Phenotype of 17-week-old WT and *pHB8:ROCK4* plants grown in a greenhouse. Bar size = 20 cm. (**c**) Plant height, (**d**) stem diameter and (**e**) number of side branches of 36-week-old poplar plants. Data shown are mean values ± SD (*n* ≥ 8). Statistical significance of differences to wild type were calculated using Student’s *t*-test. *, *p* < 0.05.

The *HB8* promoter was then coupled to the *ROCK4* gene and three independent transgenic poplar lines that express the transgene (Figure 4a) were selected for analysis. Seventeen weeks after transfer to soil pHB8:ROCK4 plants were smaller than wild-type plants (Figure 4b) with a reduction in plant height of between about 10% and 30% (Figure 4c), which is similar to the reduction seen in pIPT3:ROCK4 plants. However, in contrast to these plants, two of the three pHB8:ROCK4 lines showed a significant increase in stem diameter, of up to 25% in comparison to wild-type plants (Figure 4d). Furthermore, all three transgenic lines showed increased branching with the outgrowth of up to twelve lateral buds (Figure 4b,e). However, branch elongation was limited even in the lowest lateral branches contrasting with the stronger branching phenotype of *pIPT3:ROCK4* transgenic plants (Figure 2).

Taken together, the expression of *ROCK4* in cambial cells has led to increased radial growth, which is similar to the effect of increased cytokinin synthesis reported by [23]. However, the reduced stem elongation and bud outgrowth are not desirable traits. It could be that these growth changes are due to the mobile nature of the hormone which makes it difficult to restrict its activity to the site of gene expression. We, therefore, tested next whether a localized increase in cytokinin signaling is advantageous compared to increased cytokinin synthesis to achieve radial growth enhancement without the undesirable side effects.

### 2.3. pAHK3:ROCK3 Transgenic Plants Have an Increased Plant Height and Stem Diameter

**Figure 5:**
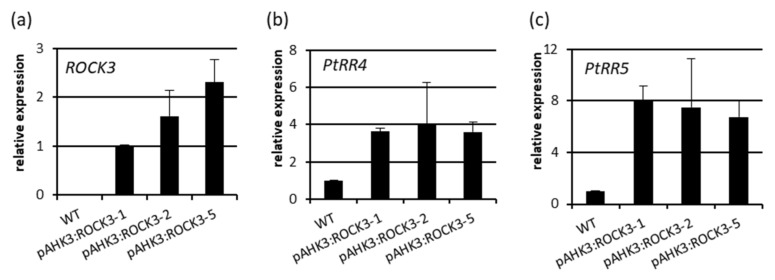
Expression of the *ROCK3* transgene and *PtRR* genes in shoots of pAHK3:ROCK3 poplar plants grown in vitro. (**a**) The relative expression of *ROCK3* and the A-type response regulator genes (**b**) *PtRR4* and (**c**) *PtRR5* were measured in stem tissue. Samples were harvested four weeks after subcultivation of plants grown in vitro. The lowest level of expression was set to 1. Data shown are mean values ± SD (*n* = 4).

We used the *ROCK3* gene, which encodes a constitutively active variant of the AHK3 cytokinin receptor of Arabidopsis [26] to increase cytokinin signaling in a cell-autonomous fashion. First, we expressed *ROCK3* under control of its original *AHK3* promoter, which is active in Arabidopsis in several tissues including meristematic zones, the vasculature and parenchyma cells [30,31]. Three independent pAHK3:ROCK3 lines expressing the transgene (Figure 5a) were selected for further analysis. Each of these lines showed four- and eight-fold higher steady state mRNA levels of *PtRR4* and *PtRR5*, indicating increased cytokinin signaling (Figure 5b,c). 

**Figure 6:**
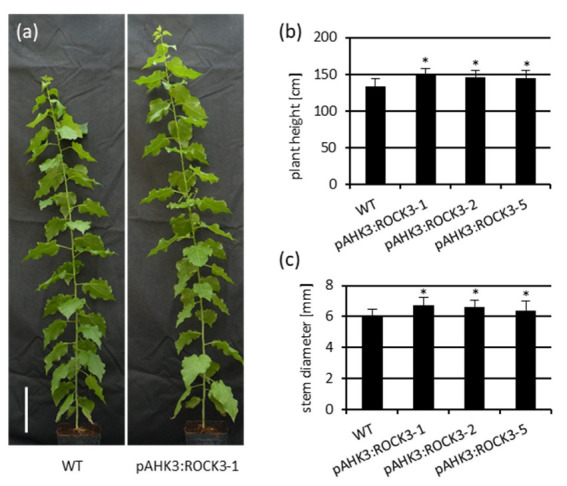
Characterization of pAHK3:ROCK3 poplar plants. (**a**) Phenotype of 13-week-old wild type (WT) and a pAHK3:ROCK3 plant (bar size = 20 cm). (**b**) Height of 13-week-old poplar plants. (**c**) Stem diameter of 13-week-old plants 20 cm above ground. Data shown are mean value ± SD (*n* = 15). Statistical significance of differences to wild type were calculated using Student´s *t-*test. *, *p* < 0.05.

Thirteen weeks after their transfer to the green house, plants of all three independent *pAHK3:ROCK3* transgenic lines showed a significantly increased plant height of about 10% in comparison to wild-type poplars (Figure 6a,b). The stem diameter of the pAHK3:ROCK3 lines was also increased as compared to wild type (Figure 6c). pAHK3:ROCK3 plants did not show the formation of additional branches or increased growth of lateral buds (Figure 6a).

**Figure 7:**
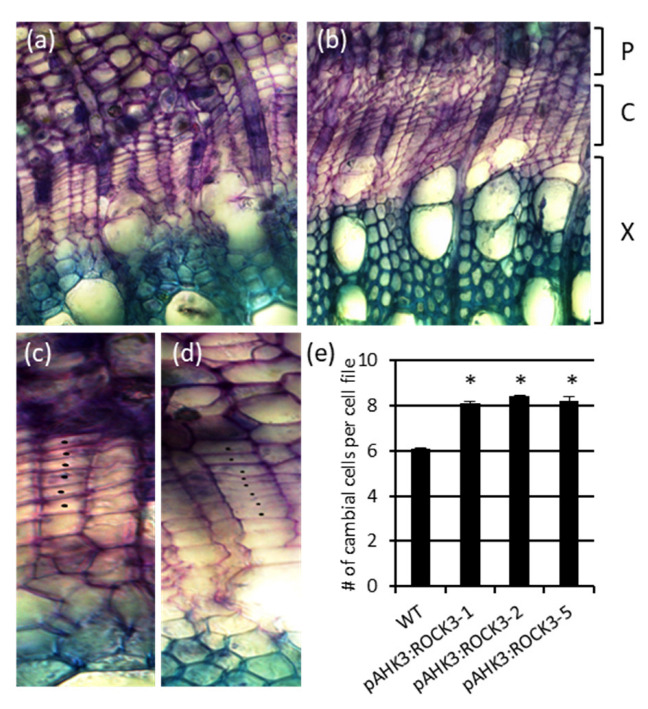
The cambial zone of *pAHK3:ROCK3* transgenic plants exhibits more cambium cells per cell file than wild type. (**a**,**c**) WT, (**b**,**d**) pAHK3:ROCK3-1. Magnification is 200× in (**a**,**b**) and 400x in (**c**,**d**). Representative pictures are shown. Black dots mark cambial cells in cell files. (**e**) Number of cambial cells per cell file. Data shown are mean values ± SD (*n* = 100). Stems of 4-month-old plants were cut 10 cm above ground. The experiment was repeated twice. Statistical significance of differences to wild type were calculated using Student´s *t*-test. *, *p* < 0.05. P, phloem; C, cambium; X, xylem.

The analysis of stem cross-sections revealed a significantly increased number of cambial cells in all *pAHK3:ROCK3* transgenic lines compared to wild-type plants (Figure 7a–d). Wild-type plants had about six cambial cells per cell file while all three transgenic lines had a significant difference of about eight cells per cell file taken at the same stem position (Figure 7e). This shows the retarded differentiation of secondary vascular tissue in the radial direction.

### 2.4. Expression of ROCK3 under Control of the HB8 Promoter

**Figure 8:**
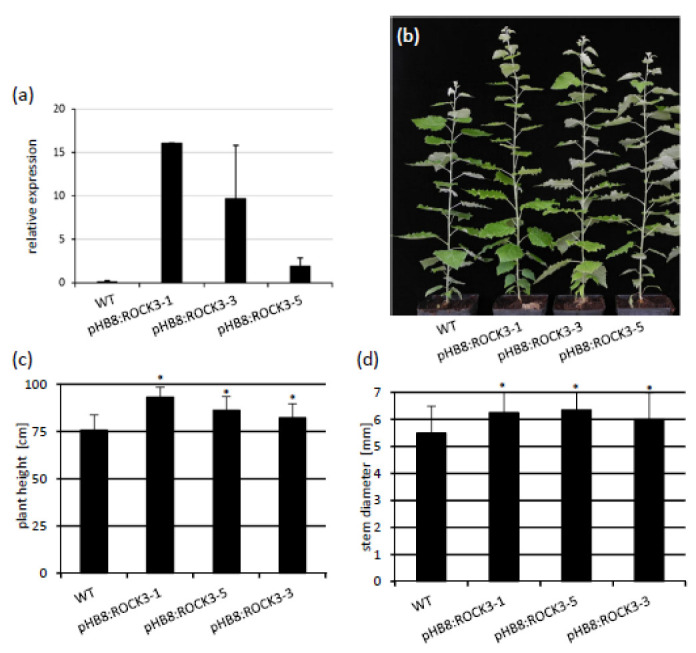
Characterization of *pHB8:ROCK3* transgenic plants grown in vitro and in a greenhouse. (**a**) Relative expression of *ROCK3* in stem tissue of poplar plants grown in vitro. Samples were taken four weeks after subcultivation. (**b**) Phenotype of 15-week-old WT and pHB8:ROCK3 plants grown in a greenhouse. (**c**) Height and (**d**) stem diameter of 15-week-old poplar plants. Data shown are mean values ± SD (*n* = 15). Statistical significance of differences to WT were calculated using Student´s *t*-test. *, *p* < 0.05.

Finally, we generated and analyzed plants expressing *ROCK3* under control of the *HB8* promoter (Figure 8a). Figure 8b shows the increased stem elongation of *pHB8:ROCK3* transgenic plants 15 weeks after their transfer to soil and growth in a greenhouse. Plants of all three transgenic lines were between 10% and 30% taller than wild-type plants (Figure 8c), and their stem diameter had increased up to 16% (Figure 8d). Taken together, this shows that restricting the expression of *ROCK3* to cambial tissue is sufficient to obtain an increase of longitudinal and radial growth.

## 3. Discussion

The comparison between the influence of an increase of cytokinin biosynthesis (ROCK4) and increased cytokinin signaling (ROCK3) on the growth of poplar plants has revealed profound differences. Increased cytokinin biosynthesis may induce an increase in radial growth, at least in the *pHB8:ROCK4* transgenic lines, but also causes additional undesired phenotypic changes such as reduced stem elongation and the premature release of lateral buds and thus increased branching. This pleiotropic phenotype is likely at least in part due to the mobile nature of the hormone, which makes it difficult to restrict phenotypic changes to the site of its synthesis [20]. In addition, it could be that the enhanced activity of ROCK4 produces supraoptimal concentrations of cytokinin. Supraoptimal concentrations of the hormone may become inhibitory for growth even in tissues such as the shoot apical meristem where cytokinin usually has a promotive activity [32]. The phenotype of *ROCK4* transgenic plants differs from that reported for *pMLX5:IPT7* plants which show an increased radial stem growth in the absence of side effects. *pMLX5* is expressed similarly to *pHB8* in the cambial zone and developing xylem [23].

An increased radial stem growth was reliably achieved by increasing cytokinin signaling cell-autonomously by ROCK3. The activity of the Arabidopsis ROCK3 receptor in poplar plants shows that it retains its enhanced cytokinin signaling capacity and couples to the poplar phosphotransmitter proteins [14] and thus the cytokinin signaling chain. The increased number of cambial cells in *ROCK3* transgenic plants indicates that cambial cells were undergoing additional divisions as compared to the wild type before entering differentiation. This is similar to the activity of cytokinin in the primary shoot apical meristem where it retards cellular differentiation as well [20,26]. In contrast to *ROCK4* transgenic plants, *ROCK3* transgenic plants showed no undesired phenotypic side-effects highlighting the advantage of the receptor´s cell-autonomous activity. 

The ectopic expression of *ROCK3* caused enhanced radial growth under the control of both promoters, the phloem-expressed *pAHK3* and *pHB8*, expressed in cambium and developing xylem. This result is consistent with the idea that cytokinin regulates cambial activity in two different ways. On the one hand, cytokinin induces the formation of a mobile signal in the phloem causing enhanced divisions in the neighboring cambial cells, and on the other hand it acts in the cambium itself [24]. The nature of the phloem-derived mobile signal is currently not known for stem tissue. In the roots of Arabidopsis a mobile cell division signal moving from the phloem to cambial cells has been identified as consisting of PEAR transcription factors [33]. Alternatively, and in analogy to cytokinin action in the shoot apical meristem through the transcription factor WUS, the hormone may act on the activity of the cambial meristem through WOX4, which is related to WUS [34,35].

The increase of radial growth in *ROCK3-* and *ROCK4*-transgenic plants is in the range reported by [23] suggesting that the consequences of an engineered enhanced cytokinin status approach the maximum of cytokinin activity. No further increase may be achieved by cytokinin as other as yet unknown factors become limiting. In any case, the increased stem growth indicates that tree biomass information is not limited by the available source strength (i.e., fixed CO_2_). Similarly, it has been shown for crop plants engineered for enhanced root growth [36] or enhanced seed yield [37] that root growth and yield were limited by sink capacity rather than by source strength. This is relevant information, as limited source strength would preclude approaches to genetically engineering the increased growth of selected tissues. 

Taken together, this study has confirmed that cytokinin promotes radial growth in poplar plants and is a rate-limiting factor. Cytokinin activity maintains cell division and retards the differentiation of meristematic cells. The comparison of enhanced cytokinin synthesis and increased cytokinin signaling has revealed a better performance of the latter. We therefore propose the *ROCK3* gene as a new tool for the genetic engineering of poplar plants to increase biomass formation.

## 4. Material and Methods

### 4.1. Plant Material and Growth Conditions

*Populus* × *canescens* (INRA 717-1B4; here called as P1) [38] was used as the wild type with the exception of *pAHK3:ROCK3* that was transformed in *Populus tremula* × *P. tremuloides* (Esch5) [39]. For in vitro propagation and shoot multiplication, plant shoots and stems were cut to approximately 10–20 mm long and placed on wood plant media (WPM) [40] supplemented with 20 g/L sucrose under sterile conditions. This propagation was repeated every four weeks. Four-week-old plantlets with roots were transferred to soil supplemented with coconut fibre (50%). Plants were grown under standard long day conditions (16 h light/ 8 h dark, 80–100 μmol m^−2^ s^−1^) in vitro in a growth chamber or in a greenhouse at 20–22 °C.

### 4.2. Gene Cloning and Transformation

All promoter-gene constructs were generated using the MultiSite Gateway^®^ Cloning Technology (Invitrogen by Life Technologies, Darmstadt, Germany). The *pAHK3:ROCK3* gene has been described [31]. To generate *pIPT3:ROCK4*, ~2000 bp of the *A. thaliana* gene *IPT3* (At3g63110) were combined with the coding sequence of the mutated gene *IPT3* named *ROCK4* [25]. Oligonucleotides used for gene amplification and cloning are listed in Supplemental Table S1. Promoters were inserted into the entry vector pDONRP4-P1R and the *ROCK3* and *ROCK4* genes were inserted into the entry vector pDONR221. Expression clones were generated by in vitro LR recombination using the destination vector pk7m24GW,3 [41]. The *pHB8* promoter was inserted in the destination vector pKGWFS7 harboring a *GFP-GUS* fusion gene as reporter [42]. Finally, constructs were transformed into *Populus* spec. using the *Agrobacterium-*mediated method with the *Agrobacterium tumefaciens* strain GV3101-pMP90RK [43,44]. After antibiotic selection, three poplar lines that originated from independent transformation events and were shown to express the transgenes were selected for analysis for each construct.

### 4.3. Morphometric Measurements

Total plant height, stem diameter (20 cm above ground) and branching behaviour of plants grown on soil in the greenhouse were determined every week beginning three weeks after planting on soil from in vitro culture. A total of 10–15 plants per line were evaluated at each time point. 

### 4.4. Histological Analysis and GUS Staining

*pHB8:GFP:GUS* transgenic plants were cut about six weeks after their transfer to the greenhouse 10 cm above ground and histological samples were taken. Cuttings were performed either by hand with a razor blade or with a Leica RM2255 microtome. Cuttings were stained in toluidine blue for various times (mostly 4–5 min), observed with an Axioskop M40 (Carl Zeiss, Jena, Germany) and digital images were taken with the associated AxioCam ICc3. Histochemical staining to detect GUS activity was performed on stem cuttings according to [45]. Thereafter endogenous pigments were removed by the incubation of cuttings in 70% ethanol.

### 4.5. Analysis of Gene Expression by Quantitative Real-Time RT-PCR

Expression of the *ROCK3* and *ROCK4* transgenes and *PtRR4* (Potri.010G037800) and *PtRR5* (Potri.001G027000) genes was quantified with total RNA extracted from the stem tuissue of plantlets cultured in vitro four weeks after subcultivation using the method described by [46]. Total RNA was treated with RNase-free DNaseI (Fermentas, St. Leon-Rot, Germany) and cDNA was synthesized from 5 µg RNA using SuperScript^TM^ III Reverse Transcriptase (Invitrogen, by Life Technologies, Darmstadt, Germany). RT-PCR was performed using the OneStep RT-PCR kit according to the manufacturer (Qiagen, Hilden, Germany). *PtACT5* (*ACTIN5*; Potri.010G204300) and *PtUBI4* (*POLYUBIQUITIN4*; Potri.001G418500) were used as reference genes. In brief, qRT-PCR was performed in a final volume of 20 µL containing 2 mM magnesium chloride, 0.1 mM of each dNTP, 0.1× SYBR^®^ Green I, 50 nM ROX, 0.01 units Immolase^TM^ DNA polymerase, and 0.2 µM of each primer (*PtUBI4*, *PtACT5*, *ROCK3*, *ROCK4*). qRT-PCR reactions comprised a hot start for Immolase^TM^ DNA polymerase activation (15 min at 95 °C), followed by 40 cycles of 15 s at 95 °C, 1 min at 60 °C, and a terminal melt curve (1 min at 95 °C, 1 min at 65 °C, 30 s at 95 °C, 15 s at 60 °C). Data were analysed with 7500 Software v2.0.1 (Applied Biosystems, Darmstadt, Germany). The primers used for qRT-PCR are listed in Supplemental Table S2.

## Data Availability

The data presented in this study are available in the article and Supplementary Data.

## Supplementary Materials

The supporting information can be downloaded at: https://www.mdpi.com/article/10.3390/ijms23158321/s1, Supplemental Table S1. Sequences of primers used for gene cloning; Supplemental Table S2. Sequences of primers used for gene expression analysis.
