# Supplementary material for: A Constitutively Active Cytokinin Receptor Variant Increases Cambial Activity and Stem Growth in Poplar
Source: Int J Mol Sci. 2022 Jul 28;23(15):8321. doi: 10.3390/ijms23158321 (PMC9369088; doi:10.3390/ijms23158321)
Supplement: Supplementary file 1 [file ijms-23-08321-s001.zip › ijms-1720617-supplementary.pdf]

Supplemental Table S1. Sequences of primers used for gene cloning.
attB sites are shown in lower case; F, forward primer; R, reverse primer

**Gene / promoter**	**Name of primer**	**Sequence (5’- 3’)**
ROCK3 gene	attB1-ROCK3-F	ggggacaagtttgtacaaaaaagcaggct ATGAGTCTGTTCCATGTGCTAGG
ROCK3 gene	attB2-ROCK3-R	ggggaccactttgtacaagaaagctgggtATGGATCCAGACGCTGTTGCAAA
AHK3 promoter	attB4-pAHK3-F	ggggacaactttgtatagaaaagttgGGACCAAGACTAGAGATACTG
AHK3 promoter	attB1-pAHK3-R	ggggactgcttttttgtacaaacttgCCACCACTTGAATACACGATCAA
ROCK4 gene	attB1-ROCK4-F	ggggacaagtttgtacaaaaaagcaggctCCATG ATCATGAAGATATCTATGG
ROCK4 gene	attB2-ROCK4-R	gggaccactttgtacaagaaagctgggtGTCACGCCACTAGACACCGCGAC
IPT3 promoter	attB4-pIPT3-F	ggggacaactttgtatagaaaagttgGACTTCGTATCTATCATGAACACT
IPT3 promoter	attB1-pIPT3-R	ggggactgcttttttgtacaaacttgTGATGAAACGCTTTGCAATATA

Supplemental Table S2. Sequences of primers used for gene expression analysis.
F, forward primer; R, reverse primer

**Gene**	**Name of primer**	**Sequence (5'- 3')**
ROCK3	ROCK3-F	TGGTCTTTTAGTTGGCTCTGTCGGT
ROCK3	ROCK3-R	CAGAAGACAACCCAAACCACCACAA
ROCK4	ROCK4-F	CCTTCATGCAAAGTCACCTG
ROCK4	ROCK4-R	CAGACAAGATACAAGTCCACCA
PtARR4	PtARR4-F	ACCAGGAGGAGAGAAGCA
PtARR4	PtARR4-R	CTAACGACGAGGGAGAACAC
PtARR54	PtARR5-F	ATGGCTGTGGAAATGGCTC
PtARR5	PtARR5-R	CAAGCCCAAAAACTCCAAGG
PtACT5	PtACT5-F	CATTGGTGCTGAGCGATTCCGTTGC
PtACT5	PtACT5-R	TTTTCATGCTGCTTGGGGCTAGTGC
PtUBI	PtUBI-F	GTTGATTTTTGCTGGGAAGC
PtUBI	PtUBI-R	GATCTTGGCCTTCACGTTGT
